# Informational Entropy Threshold as a Physical Mechanism for Explaining Tree-like Decision Making in Humans

**DOI:** 10.3390/e24121819

**Published:** 2022-12-13

**Authors:** Javier Cristín, Vicenç Méndez, Daniel Campos

**Affiliations:** 1 Istituto Sistemi Complessi, Consiglio Nazionale delle Ricerche, UOS Sapienza, 00185 Rome, Italy; 2 Dipartimento di Fisica, Universita’ Sapienza, 00185 Rome, Italy; 3 Grup de Física Estadística, Departament de Física, Facultat de Ciències, Universitat Autònoma de Barcelona, 08193 Bellaterra, Barcelona, Spain

**Keywords:** decision making, entropy, eye-tracking

## Abstract

While approaches based on physical grounds (such as the drift-diffusion model—DDM) have been exhaustively used in psychology and neuroscience to describe perceptual decision making in humans, similar approaches to complex situations, such as sequential (tree-like) decisions, are still scarce. For such scenarios that involve a reflective prospection of future options, we offer a plausible mechanism based on the idea that subjects can carry out an internal computation of the uncertainty about the different options available, which is computed through the corresponding Shannon entropy. When the amount of information gathered through sensory evidence is enough to reach a given threshold in the entropy, this will trigger the decision. Experimental evidence in favor of this entropy-based mechanism was provided by exploring human performance during navigation through a maze on a computer screen monitored with the help of eye trackers. In particular, our analysis allows us to prove that (i) prospection is effectively used by humans during such navigation tasks, and an indirect quantification of the level of prospection used is attainable; in addition, (ii) the distribution of decision times during the task exhibits power-law tails, a feature that our entropy-based mechanism is able to explain, unlike traditional (DDM-like) frameworks.

## 1. Introduction

In our daily life, we constantly find ourselves in situations that imply making decisions: “What I am going to eat, which film will I see, or will I be on time for the next bus?”. In all of these situations, we need to evaluate the different options available as a way to elucidate the best one. While exploring such situations would lie within the field of psychology, in recent years, there has been a growing interdisciplinary interest in decision making. Determining the neural correlates of decision mechanisms constitutes an important subject in cognitive and behavioral neuroscience [1,2,3,4]. In addition, the mathematical study of decision strategies and their comparison with subjects’ performance represents an important subject in game theory and econophysics [5,6]. Last but not least, ideas from statistical physics and complex systems have also made their way into the field. While most contributions to date focus on decision making at the level of groups or collectives (see [7,8,9,10,11] for some reviews), tentative works suggesting physical principles that could be involved in individual decisions also exist [12,13,14,15,16].

Up until now, large efforts have been made to understand the dynamics and characteristics of perceptual decisions, that is, those in which sensory information provides direct evidence about what the correct option is, such as in the famous random dot motion task [17,18]. So, a correspondence between such sensory information and the neuronal responses responsible for the accumulation of evidence in the brain is assumed to be identifiable in some way. Alternatively, value-based or preferential decision making involves situations in which a deliberative and subjective (up to a certain level) process is necessary to reach a decision, such as when a subject is asked to choose between two food items. In such cases, neural correlates obviously become more difficult to identify.

A different class of decision making corresponds to the case in which an objectively correct option does exist, but this option cannot be trivially identified from sensory information alone because successively coupled decisions arranged in a tree-like fashion are involved. Following some of the existing literature (see [19,20]), we will denote this as *sequential* decision making. This requires a higher cognitive capacity and a slower and more reflective response by the subject in order to process the information. Hence, these situations are essentially restricted to humans (and maybe some other higher organisms). They include tasks such as playing board games, such as chess, or solving mazes or tasks presented in some intelligence tests. All of these examples involve decisions in which a tree-like structure of future possibilities must ideally be built by the subject. In the present work, we will use the term *prospection* to denote such hypothetical, or mental, simulations of future events [21,22,23].

For the case of perceptual decision making, most theoretical frameworks that aim to explain the underlying dynamics lie within the *accumulator* framework. According to this, cognitive evidence (described through some effective stochastic variable) is gained over time until it reaches a given threshold, which then triggers a decision. The paradigmatic example is the drift-diffusion model (DDM) [24], where the relative evidence in favor of the different options is assumed to follow a Brownian diffusion process, with a drift that accounts for the trend towards the correct option. Nowadays, it is widely accepted among psychologists that the success of the DDM is overwhelming [25,26], though in many cases, this requires non-trivial modifications or extensions, such as time-dependent thresholds [27] or dynamic changes in the drift [28]. Furthermore, recent works have shown that value-based decisions can also be accommodated within this framework provided that the thresholds are assumed to progressively collapse over time [29,30].

On the contrary, stochastic mechanisms that are able to capture the dynamics for sequential decision making are much less frequent due to their complexity (see [31,32,33] for some significant exceptions). Here, we will provide experimental evidence that these processes in humans are compatible with a stochastic framework in which computational information (computed through Shannon’s entropy) may be implicitly computed by the individual as a mechanism for assessing the uncertainty about options before making a decision. To illustrate this, we studied the performance of subjects during a particular task of navigation through a maze on a computer screen in combination with eye-tracking data to assess the corresponding behavioral dynamics. We did not introduce any explicit costs for prospecting or analyzing information, as there were no time constraints present in the task. Thus, we posed an extreme situation in which decisions were mostly driven by the optimization of the prospection process.

In Section 2, we will present our information-based framework and discuss its main conceptual differences from accumulator models that are used for perceptual decision making. In Section 3, we will show our experimental results to describe the performance of the subjects in the navigation task. A comparison of this performance with that shown by virtual (random-walk) algorithms that are able to prospect information ideally allows us to infer the level of information that humans really process during the task. This reveals that human performance can only be explained if prospection is actually being used in the task, and we can even quantify that level of prospection. Next, we explore the statistical properties of the decision times observed during the task to provide quantitative evidence that human performance is compatible with the entropy-based mechanism proposed here. The conclusions from these results are then discussed in Section 4, and the experimental and numerical methods employed for the analysis are detailed in Section 5.

## 2. Theoretical Framework

A relevant problem in decision making is the establishment of a criterion for identifying when we have enough information to discriminate between alternative options, e.g., options *A* and *B* for a binary decision. This can be accounted for by sequential analysis. Let $x_{n}=\left\{x_{1},x_{2},\ldots ,x_{n}\right\}$ be a set of independent samples or observations that provide some information about the options. Then, we want a termination or stopping criterion to determine when $x_{n}$ provides a sufficient level of evidence in favor of (or against) one of the options.

A famous solution to this problem is the Sequential Probability Ratio Test (SPRT)—originally developed by Abraham Wald [34]—which minimizes the size *n* of the set required to accept or reject one of the options with a fixed level of reliability. Given $x_{n}$, we can map all of its information into the cumulative probabilities $p_{A,n}$ and $p_{B,n}$, which we assign to options *A* and *B*, respectively (with $p_{A,n}=1-p_{B,n}$ if the two options are mutually exclusive). The SPRT criterion establishes that a decision can be reliably made as soon as the cumulative log-likelihood function

(1) $$W_{n}=\ln\left(\frac{p_{A,n}}{p_{B,n}}\right)$$

exceeds (or falls below) a given threshold $W_{\mathrm{th}}$. Consequently, the SPRT criterion establishes that there is a minimum amount of evidence required to decide. The DDM can be seen as a particular continuum implementation of the SPRT [34,35]. In controlled experiments of perceptual decision making, the sample set $x_{n}$ corresponds to direct sensory evidence that is mapped into the probabilities $p_{A,n},p_{B,n}$ in a relatively easy manner. For example, $x_{n}$ can typically account for visual evidence in favor of one of the options.

### 2.1. Entropy Refinement

In a sequential decision-making context, the existence of a mapping between the sensory evidence and the probabilities $p_{A,n},p_{B,n}$ is far less obvious. Gazing at one of the options, for example, does not necessarily translate into an increase in its probability.

**Figure 1:**
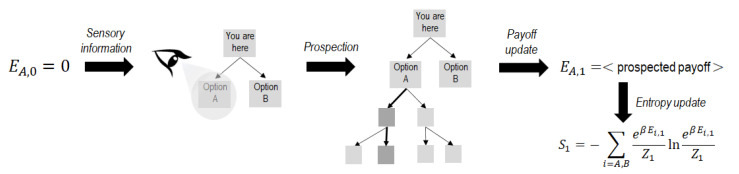
Scheme of the mechanism proposed for prospection and evaluation of updates during sequential decision making. Starting from no a priori payoffs for any option, the sensory information determines the region of the decision tree that is being prospected by the individual (option A in the example shown). Prospected paths (path deriving from option A that is emphasized) lead to a first update of the payoff $E_{A,1}$ given by the average payoff that would result from the paths prospected. These payoffs are then used to evaluate the cumulative Shannon entropy by using canonical probabilities (see the text for details).

To overcome this difficulty, here, we hypothesize that the option that is being observed by the subject is being used to assess the decision tree that would result from choosing that option. That is, we assume that sensory evidence provides information about the prospection process that the subject is performing. The mechanism that we propose for this works as follows. Using, as above, the binary example for illustration, we define $E_{A,n}$ and $E_{B,n}$ as the payoffs estimated from that prospection process after the sensory information $x_{n}$ has been gathered. We initialize the system by setting $E_{A,0}=E_{B,0}=0$ (no a priori payoffs are assigned to any option). Then, sensory-evidence accumulation begins through a first sample $x_{1}$ (e.g., the individual starts by prospecting option A, and so a set of the possible future paths starting from option A are analyzed). The corresponding $E_{A,1}$ is updated as the average payoff that would result from following the paths prospected (see Figure 1).

As a second assumption, we will consider that the updated payoffs $E_{A,n}$ and $E_{B,n}$ are interpreted by the subject as an estimation of their actual average values (so correlations or higher-order statistics in the payoffs are internally neglected by the subject). Following the prescriptions from the maximum entropy principle (MEP) [36], if the only information available from a set of stochastic variables (the payoffs $E_{A,n}$ and $E_{B,n}$, in this case) is their average values, then the most neutral, or unbiased, choice of a probability map $p_{i,n}=p_{i,n}\left(E_{i,n}\right)$ that one can build is

(2) $$p_{i,n}=\frac{e^{\beta E_{i,n}}}{Z_{n}},$$

where β is a positive constant (which appears as a Lagrange multiplier when applying the formalism of the MEP) and $Z_{n}$, a normalization factor for guaranteeing that $\sum_{i}p_{i,n}=1$.

According to all of this, we will consider that the Boltzmann-like distribution (2) is used by subjects to map payoffs into the probabilities $p_{A,n}$ or $p_{B,n}$ as a result of the prospection process (see Figure 1). Within this hypothesis, $\beta^{-1}$ represents a characteristic measure of how different the payoffs for option A or B should be to yield significantly different probabilities for the two options.

Finally, we need to introduce a termination or stopping criterion by which the decision will be triggered. We argue that a plausible mechanism for sequential decisions must be based on assessing the amount of information that the probability distribution (2) contains. The most direct way to compute such information is, obviously, Shannon’s entropy $S_{n}=-\sum_{i}p_{i,n}\log\left(p_{i,n}\right)$ (with i=A,B for binary decisions). As a result, we propose the following termination rule:

- If $S_{n}>S_{\mathrm{th}}$: continue prospecting;
- Else: accept the option *i* satisfying $\max_{i}\left[p_{i,n}\right]$.

That is, a threshold $S_{\mathrm{th}}$ in the Shannon entropy is introduced to trigger the decision in favor of the most likely option at that moment. At this point, we remember that Shannon’s entropy reaches its maximum value when no information is still available (so $p_{A,0}=p_{B,0}$), and its value decreases as long as greater evidence in favor of one particular option is gained according to the mechanism specified in Figure 1.

**Figure 2:**
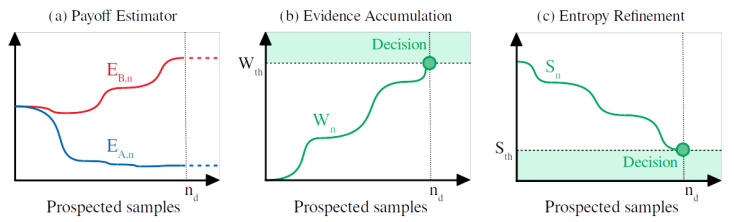
Scheme for the accumulator and reliability mechanisms. (**a**) Payoff estimation during successive prospected samples *n*. (**b**) Evolution of Wald’s ratio $W_{n}$ according to the payoff estimators (the decision is made at $n_{d}$ when $W_{n}$ reaches the threshold $W_{\mathrm{th}}$). (**c**) Evolution of Shannon’s entropy $S_{n}$ according to the payoff estimators (the decision is made at $n_{d}$ when $S_{n}$ reaches the threshold $S_{\mathrm{th}}$).

So, the *evidence accumulation* mechanism typically associated with the DDM is here replaced with an *entropy refinement* mechanism (see Figure 2). Actually, we note that this idea is not completely novel, but other authors in the literature have previously discussed similar mechanisms [37,38,39]. Interestingly, we note that combining (1) and (2) leads to $W_{n}=\beta \left(E_{A,n}-E_{B,n}\right)$, so the SPRT can be interpreted, under the mapping (2), as a termination criterion based on a threshold in the difference between the estimated payoffs.

### 2.2. Working Example

Before testing our ideas against experimental data, though, we will illustrate some properties of the *entropy refinement* mechanism (ERM) through an idealized working example. Our intention is to identify some relevant and measurable differences in the decision-making process that allow us to discriminate between the ERM and the traditional approaches based on the DDM or the SPRT.

If a subject has to choose between options *A* and *B* (whose actual payoffs read $\mu_{A}$ and $\mu_{B}$, respectively), this will be done by successively sampling/prospecting information from the two options to obtain the estimates $E_{A,n}$ and $E_{B,n}$ (with *n* again representing the number of samples). For simplicity, we assume that every sample *i* provides a new piece of information $\epsilon_{A,i}$ and $\epsilon_{B,i}$ about options A and B, respectively, which can be represented as stochastic Gaussian variables with means $\mu_{A}$ and $\mu_{B}$, as well as the unit variance. So, the information obtained provides an approximation of the actual values $\mu_{A}$ and $\mu_{B}$, and the estimated payoffs can be computed through the averages $E_{A,n}=\frac{1}{n}\sum_{i=1}^{n}\epsilon_{A,i}$ and $E_{B,n}=\frac{1}{n}\sum_{i=1}^{n}\epsilon_{B,i}$. This is in agreement with our assumption above that an individual essentially uses prospection to obtain an averaged estimation of the actual payoffs.

Once we have $E_{A,n}$ and $E_{B,n}$, we can compute (using (2)) the corresponding Shannon entropy and determine whether a particular threshold $S_{\mathrm{th}}$ is reached to trigger the decision or go on with the information sampling instead.

The main magnitude that we will explore is, as usual, the statistics of the decision times, that is, the number of samples *n* required to trigger the decision. Note that many works on decision making focus on the average values of the decision time, or, alternatively, histograms of the decision time are fitted to gamma distributions [40,41]. However, to discriminate between the ERM and an accumulator (DDM-like) mechanism, here, we will rather focus on exploring the behavior at the tail of the probability distribution of decision times. Previous works based on ideas similar to that of the ERM have suggested that this mechanism can account for power-law distributions of decision times [42,43], so this can represent a significant difference from other mechanisms in which such distributions often decay exponentially.

Accordingly, we carry out numerical experiments by using the rules above and compare the distributions of decision times for the ERM and for an accumulator scheme that, as in the SPRT, uses the fact that the difference between the payoffs $\left|E_{A,n}-E_{B,n}\right|$ reaches a threshold $W_{\mathrm{th}}$ as a termination criterion instead.

**Figure 3:**
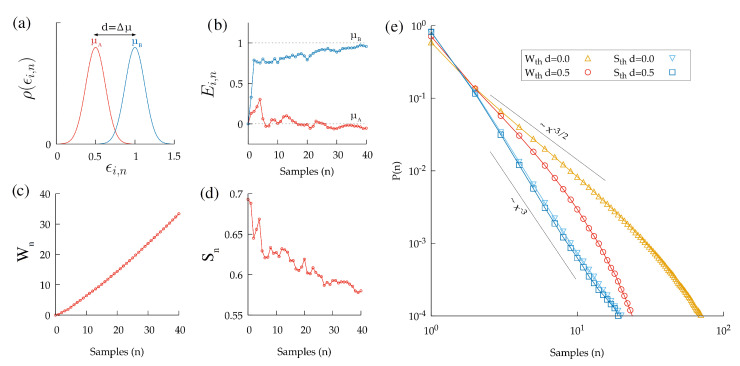
(**a**) Probability distributions for the stochastic variables $\epsilon_{i,n}$ (with *n* being the number of samples and where *i* labels the options *A* and *B*). The means $\mu_{A}$, $\mu_{B}$ represent the actual payoffs for each option. (**b**) Evolution of the estimator $E_{i,n}$ as a function of the number of samples *n*. (**c**) Evolution of the cumulative $W_{n}$ with the number of samples *n* and with β=1. (**d**) Evolution of the Shannon entropy $S_{n}$ with the number of samples *n* and with β=1. (**e**) Probability distribution for the number of samples to reach $S_{\mathrm{th}}$ or $W_{\mathrm{th}}$ for the ERM and the SPRT, respectively, and for different distances $d\equiv \mu_{A}-\mu_{A}$. We simulated $10^{7}$ decisions to obtain these distributions. The thresholds were set to $S_{\mathrm{th}}=0.5$ and $W_{\mathrm{th}}=0.25n$.

The results obtained are illustrated in Figure 3 as a function of the values of $\mu_{A}$ and $\mu_{B}$ and of the thresholds $W_{\mathrm{th}}$ and $S_{\mathrm{th}}$. In summary, we find that the SPRT exhibits a decision time distribution that depends strongly on the distance between the means of the payoffs $d\equiv \mu_{A}-\mu_{B}$ (Figure 3e), and for most situations, it eventually decays exponentially (though transient power-law behaviors with exponents of −1.5 are also found). Instead, for the ERM, the distribution exhibits a power-law behavior $P\left(n\right)\propto n^{-3}$ for a wide range of situations. Remarkably, the power-law behavior with the −3 exponent persists when considering decisions between more than two options; in the Supplementary Materials, we show equivalent results for decisions between four possible options.

So, at this point, we have at least one qualitative difference that we can use to discriminate between the SPRT and the ERM.

## 3. Experimental Results

**Figure 4:**
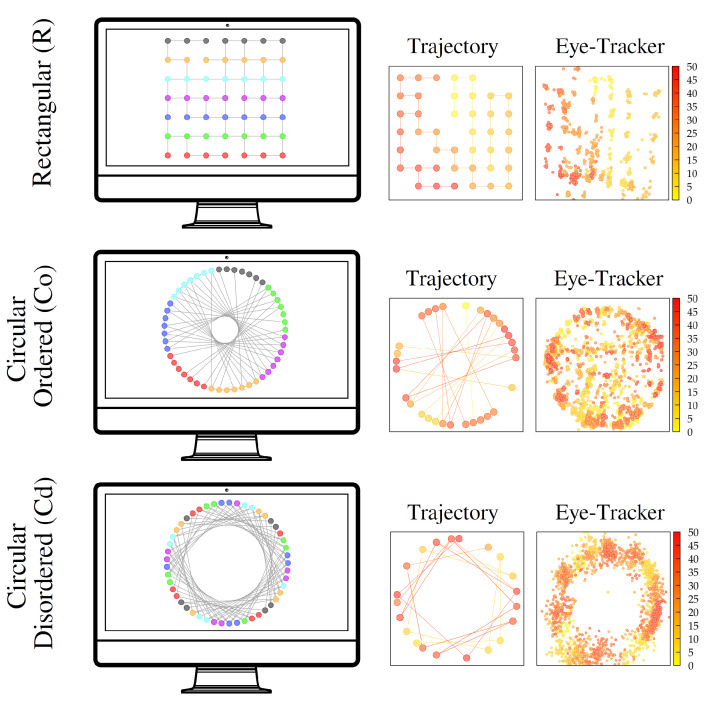
Scheme of the experimental setup. First column: Visualization of the 49-node lattice used for the navigation task. The solid lines indicate the bonds allowed between neighbor nodes. Second column: A realization of an individual trajectory within the lattices (the color code denotes the time; see the legend). Third column: Eye fixations obtained during the previous trajectory from eye-tracking data.

We designed a particular task of navigation through a maze on a computer screen, where a correspondence between sensory (visual) information and prospection could be reasonably expected. Subjects were asked to visit the maximum possible number of nodes of a discrete lattice containing 49 nodes in 49 moves while taking into account that moves were only possible between nodes connected through bonds (marked as lines; see the left column in Figure 4). So, they had to progressively develop a strategy to reach regions/nodes of the lattice that remained unexplored. Moves were carried out by clicking with the mouse on the node to which one wanted to move next. To explore the subjects’ performance in the task under different levels of difficulty, three different visual representations were used (rectangular, circular ordered, and circular disordered; see the left column in Figure 4). However, all of the structures presented to the subjects were topologically identical in order to facilitate the comparison of the results; only the visual representation changed from one to another. Further details about the experimental design and protocol are provided in Section 5.

Connecting the experiment with our theoretical framework, every move from one node to another was considered as a single decision, and the nodes observed before a decision were considered as the successive information samples $\left\{x_{1},x_{2},\ldots ,x_{n}\right\}$ that the subject was gathering. Finally, the estimated payoffs $E_{i,n}$ (with *i* representing the specific options available) would be taken as the average number of newly visited sites that would result from following the paths prospected.

Commercial eye trackers were used during the task to determine where the subjects were gazing. From that information, we inferred the possible future paths and decision trees that the subjects were mentally exploring. While we could not know the specific paths that the human subjects were prospecting (or whether they were really prospecting) directly from the eye-tracking data, we used the fraction of time for which the participants gazed at regions of the lattice as a proxy for this. So, we assumed that the number of prospected paths related to choosing one specific option was proportional to the time for which the subject gazed at that particular option (see Section 5.2 for details).

### 3.1. Overall Performance in the Navigation Task

**Figure 5:**
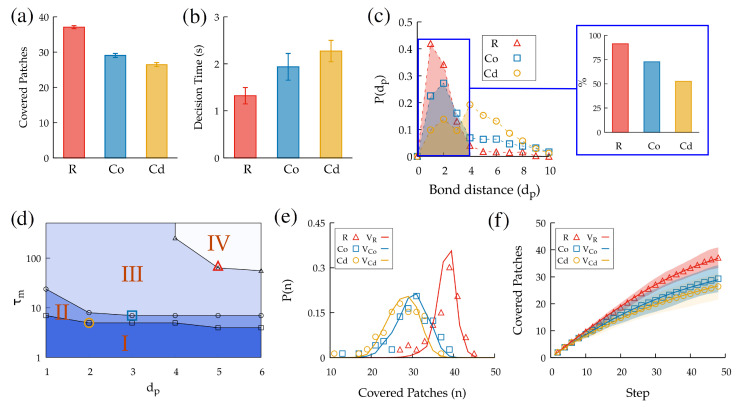
(**a**) Performance of human subjects in the task for the three levels of visual organization presented in Figure 4. (**b**) Averaged decision times for the three levels. (**c**) Distribution of the distance $d_{b}$ between the current node and nodes gazed at between moves (inset: cumulative probability that the nodes gazed at satisfy $d_{b}\leq 4$). (**d**) Performance of a virtual walker in comparison with the experimental ones, with regions II, III, and IV accounting for virtual walker performances that were better than those of humans in the R, CO, and CD cases, respectively. (**e**) Best fit (lines) for the experimental distribution of performances (symbols) obtained from the virtual walker algorithm (see the text for details of the fit). (**f**) Evolution of the performance during the 49-move trajectories obtained from the experimental trajectories (symbols) and the virtual walkers with the best-fit parameters (lines). The virtual walker trajectories corresponded to the average behavior after performing $10^{3}$ simulations. The colored region corresponds to the standard error of those simulations.

The overall performance of the individuals was computed as the number of nodes that a subject was able to cover during the entire trajectory of 49 moves (Figure 5a). For the rectangular level, the subjects visited an average of 37.1±3.8 nodes (that is, 75.7% of the total of 49 nodes). For the circular-ordered level, they covered 29.1±4.8 nodes (59.4%), and for the circular-disordered graph, they covered 26.4±4.8 nodes (53.9%). These results confirm that the navigation task (and, thus, the sequential decisions involved) largely depended on the visual representation of the nodes in the lattice, with more complex representations preventing the subjects from planning their trajectories ahead of time (thus suppressing or reducing prospection). Furthermore, analyzing the performance as a function of the averaged decision time showed us that a higher performance was not a result of spending more time before deciding (Figure 5b), but the difficulty of the task seemed to be the main reason for this (note that the decision time is here defined as the time between consecutive moves).

### 3.2. Eye-Tracking Data Captured Prospection Dynamics

We next analyzed the information gathering during the task with the help of the eye-tracking data. We define the distance $d_{b}$ as the minimum number of moves required to go from the current node of the lattice to the one at which an individual is gazing. The probability distributions of this variable were again found to be completely different for the three levels of visual organization (Figure 5c). Then, it was clear that the individuals could not prospect equally in the three cases. While for the rectangular level, a large amount of time was invested in gazing at nearby nodes, for the two circular levels (especially for the disordered one), frequent gazes at distant nodes were observed. These must be attributed either to (i) distractions caused by the presence of nodes that were close on the screen configuration, though they were not easily accessible from the current one, or (ii) the difficulty in easily identifying the nodes that would be available in the next few steps. Ideally, an efficient prospection of the future paths should combine an intensive exploration of closer nodes and a smaller (but non-negligible) exploration of further ones. We illustrate this in the inset of Figure 5c, where the cumulative probability of gazing at nearby nodes (defined as those with $d_{b}\leq 4$) is shown to drastically decrease as a function of the visual difficulty of the task.

### 3.3. Quantifying Prospection during Navigation

As a way to quantify and refine the ideas above, we compared the subjects’ performance in our task to that of virtual subjects that followed an algorithm that was able to automatically prospect all of the information of the paths available within a certain number of moves $d_{p}$ (called the *prospection length*). So, the extreme case $d_{p}=0$ would correspond to a subject that was not able to prospect any information and, thus, carried out a *blind* random walk through the lattice; a virtual subject with $d_{p}=1$ would only be able to discriminate whether first-neighbor nodes had been visited in the past or not, and so on. As $d_{p}$ increased, these virtual subjects (walkers) then had the ability to avoid their own previous paths in order to avoid revisits to those nodes. Using the number of unvisited sites available to compute the payoffs $E_{j,n}$ and computing the move probabilities through (2), we then used rules that were equivalent to standard models of self-avoidance in statistical physics, such as in the true self-avoiding random walk [44,45,46] and the self-attracting random walk [47,48,49]. The implementation details for these rules are provided in Section 5.

To increase the level of realism of these virtual walkers and facilitate the comparison with the experimental data, we additionally considered that they were only able to keep in memory whether a particular node had been visited or not for a characteristic time $\tau_{m}$. For large values of $\tau_{m}$, the memory remained unaltered, and so all visited sites were remembered, while for small values of $\tau_{m}$, nodes that had been visited in the distant past were forgotten.

We compared the performance of the virtual and human subjects to infer the prospection abilities that were presumably being used by the human subjects in the experiment as a function of the level of the visual organization/representation. In particular, by exploring a reasonable range of $d_{p}$ and $\tau_{m}$ values in the algorithm, we observed that the parameter phase space could be divided into four regions (see Figure 5d). For region I, the algorithm produced an average number of visited nodes that was lower than that of the individuals in any of the experiments. Region II produced a performance that lay between the results obtained for circular ordered and circular disordered. Region III overcame the results for the circular-ordered performance, but not for the rectangular performance. Region IV, finally, outperformed all of the experimental results.

Hence, we concluded that relatively large values of both $\tau_{m}$ and $d_{p}$ were necessary for the virtual walkers to equal or improve the performance of the human subjects in the rectangular level. This confirmed that the subjects in this case remembered the previously visited nodes during the task and efficiently predicted future paths. The prospection ability, in particular, is indispensable for justifying the performance seen in the experiments. Instead, for the circular structures, the individuals were probably not able to prospect the paths to distant nodes (information gathering was less efficient, as suggested before in Figure 5c); in consequence, the value of $d_{p}$ necessary to reproduce their performance was not necessarily high (though some level of memory $\tau_{m}$ was still necessary). In the Supplementary Materials, we explore the case in which $d_{p}$ was not a fixed value, but followed a certain probability distribution, and for that case, our conclusions remain unaltered.

Next, we determined the values of $d_{p}$ and $\tau_{m}$ that provided the best fit to the distribution of performances obtained from the experiments (see Figure 5e). These were (i) $\tau_{m}^{R}=70$, $d_{p}^{R}=5$, (ii) $\tau_{m}^{Co}=7$, $d_{p}^{Co}=3$, and (iii) $\tau_{m}^{Cd}=5$, $d_{p}^{Cd}=2$, for the rectangular (R), circular-ordered (Co), and circular disordered (Cd) levels, respectively.

From this, we analyzed the evolution of the performance throughout the task between humans and the virtual walkers with the fitted parameters (Figure 5f). The performance increased almost linearly in the beginning (where avoiding visited nodes was relatively easy), but the growth slowed down as time advanced and trajectory overlaps appeared. The experimental curves (symbols) and those obtained from the virtual walkers (lines) with the fitted parameters agreed almost perfectly. This is an indirect confirmation that the behavior of virtual walkers with prospection was able to accurately reproduce the dynamic performance of human subjects throughout the experiment.

### 3.4. Human Decisions during Maze Navigation Are Compatible with the ERM

**Figure 6:**
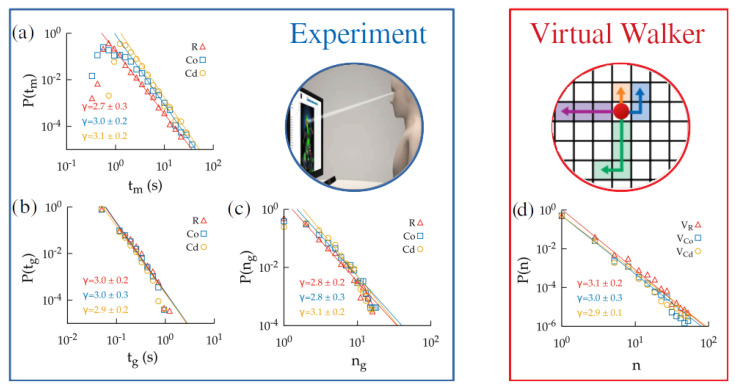
Distributions of time between consecutive moves $t_{m}$ (**a**), the time spent gazing at a given node $t_{g}$ (**b**), and the number of patches gathered between consecutive movements $n_{g}$ (**c**) obtained from the experimental data. (**d**) Distribution of the number of prospections *n* performed by a virtual random walker with $S_{\mathrm{th}}=0.5$. In all cases, the exponent obtained from a power-law fit to the distributions is highlighted, with the different colors representing the difficulty levels of R, CO, and CD.

The working example explored in Section 2 yielded a power-law scaling (with exponent −3) for the tail of the decision time distributions within the ERM framework. Actually, this result is not specific to that particular example (based on Gaussian estimations of the actual payoffs). Using the virtual random walks with prospection described in the previous section, we obtained exactly the same behavior (Figure 6d) with a wide range of parameter values for $d_{p}$, $\tau_{m}$, and $S_{\mathrm{th}}$, so we can infer that this represents a rather general property of the proposed ERM mechanism (in the Supplementary Materials, a study of robustness is carried out to check that this result does not critically depend on the parameter choices in the model).

To check if the performance of the human subjects in the navigation task also showed the same scaling, we used the eye-tracking data from the experiments to analyze the distributions of (i) the time between consecutive moves in the experiment, $t_{m}$, (ii) the time during which the subjects gazed at the same patch, $t_{g}$, and (iii) the number of different nodes gazed at before making the next move, $n_{g}$. The first value would represent our best estimation of the decision times in the experiment, while the other two were also provided as alternative measures for the sake of completeness.

The results found showed consistent evidence in favor of a power-law scaling with an exponent close to −3 for the three cases of $t_{m}$, $t_{g}$, and $n_{g}$ (Figure 6a–c). It is especially remarkable that the results obtained for the three levels of visual organization (rectangular, circular ordered, and circular disordered) were the same, despite the human performance in these three cases being clearly different (Figure 5). This suggests that a common underlying mechanism for decision making was used by the subjects in the experiments, though their different levels of difficulty led to differences in the performance. While the time range over which the power-law scaling extended was not very wide (since the decision times in the experiment only spanned two orders of magnitude), the fits were quite robust; only longer decision times (for which statistics were not very significant, since very few decisions extended over so much time) showed significant departures from it. Furthermore, we remark again that SPRT frameworks often predict gamma distributions of decision times with exponential decays, so they would be unable to explain these results.

### 3.5. Information Statistics at the Moment of the Decision

**Figure 7:**
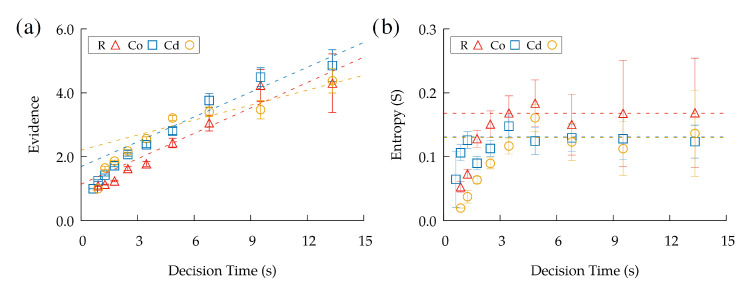
(**a**) Maximum relative evidence between the options at the moment of the decision. Linear fits (for times larger than 3 s) are given by f(x)=0.26x+1.14 (R), g(x)=0.26x+1.69 (CO), and z(x)=0.16x+2.21 (CD). (**b**) Shannon’s entropy $S_{n}$ at the moment of the decision. The horizontal lines correspond to the averaged entropy for times >3 s (0.168 (R),0.131 (CO), and 0.130 (CD). A statistical test is given for the null hypothesis that the entropy was non-constant for times >3 s. The corresponding *p*-values are p=0.82 (R), p=0.69 (CO), and p=0.92 (CD).

As mentioned above, the SPRT criterion with canonical probabilities (2) is equivalent to assuming that a decision is triggered once the payoff difference $\left|E_{A,i}-E_{A,i}\right|$ reaches a given threshold. Our data clearly show that this estimator, if computed from the experimental data at the moment of making the decision/move, increases monotonically with the time that is necessary to make the decision. So, longer decisions involve longer evidence accumulation (Figure 7a), which is in clear contradiction with the criteria of the SPRT.

Instead, when plotting the Shannon entropy (computed from the procedure above) at the moment of the decision, for long decision times, it tended to a value that was approximately constant. The statistical significance of this result was verified by testing the null hypothesis that the entropy was non-constant (see the figure’s caption for details). In addition, we have checked that the statistics of decisions did not vary significantly between the first moves and last moves of the trajectory, so the idea that our results were due to non-stationary effects in the task can be discarded (see the Supplementary Materials).

Hence, *S* could be reasonably considered as a trigger of the decision, at least for longer decisions (Figure 7b). Shorter decisions (<2 s), instead, were probably induced by an automatic response by the subjects, who sometimes planned their decisions ahead for multiple steps and, thus, moved to the next node according to prior information that was already gathered during the previous move. We stress that the −3 power-law scaling discussed above was essentially obtained for longer decision times in the same range, too. So, it seems reasonable that our model essentially captured the moves made at the instants in which information acquisition was carried out, but not subsequent moves that were made automatically by the subjects.

## 4. Conclusions

Navigation efficiency in higher organisms (humans, in particular) must take into account the fact that they are able to prospect the future outcomes of their available options and process the corresponding information in order to reach a decision. Here, we explored this idea within the context of human navigation through mazes in which non-local information was available through visual inspection (and, thus, information was processed in a tree-like fashion prior to the decision).

Our analysis (based on comparing the performance of human subjects with that of virtual walkers with the ability to prospect future paths) provided evidence that prospection was necessarily being used by humans, at least in the levels of visual organization that enabled it (especially in the rectangular one). In addition, an approximate quantitative characterization of that prospection capacity ($d_{p}$) and the associated memory skills ($\tau_{m}$) was obtained, thus reaching an estimation of the quantity of information that the humans were really managing during the task.

Furthermore, the distribution of time between moves—or gazing time—together with the study of the values for the entropy at the moment of the decisions allowed us to think that the ERM can account for how information is being processed by the subjects during the task to a significant extent, especially for (longer) decisions that are made after a subject decides to stop and gather new information. In this respect, we stress that, traditionally, the mean time taken to make a decision, as well as the ratio of the time corresponding to choosing option *A* or *B* (for binary decisions), has been studied in detail by psychologists. On the contrary, the tails and the details of the decision time distributions are rarely explored in decision-making experiments. Here, we have shown that such statistical details can provide very significant information about the dynamics of decisions that are being used.

Regarding the −3 value of the power-law exponent found for the ERM formalism and from the experiments, a formal justification of its origin remains to be found. For the specific navigation task studied here, decision times must be understood as the sum of the time that an individual had gazed at each node before making a new move. Then, to explain the power-law scaling, one should argue that either (i) the distribution of time for which the subject kept looking at a given patch or (ii) the number of patches that were gazed at between decisions must have power-law tails. It is, however, the case that both distributions present that scaling (see Figure 6a,b). So, the underlying mechanism yielding the power-law distribution for decision times is apparently a non-trivial combination of both. It is still not clear how general these results may be, or if they appear as a consequence of the specific conditions in our experiment. However, we stress that similar results have also been found in other experiments of human navigation through mazes [50], so, all together, this raises the need for a deeper and more systematic exploration of these ideas in the future.

Finally, it is remarkable that all of this information about sequential decision making in humans was obtained simply with the help of eye-tracking data and the monitoring of the decision time exhibited by subjects on a computer screen, which required only easily available technologies. It is likely that the combination of such methods and data with EEG or other advanced physiological sensors could be used to refine our ideas and provide more reliable estimates of the dynamics during sequential tasks. We hope that our results can stimulate further research in this line.

## 5. Methods

### 5.1. Experimental Design

A total of 18 clinically normal adults (10 women and 8 men) aged from 18 to 45 carried out the experiment. Informed consent was obtained from all participants. All experimental protocols were approved by the Universitat Autònoma de Barcelona and by its ethics committee. All experiments were carried out in accordance with the guidelines and regulations that were applied at that time by the Catalan and Spanish Governments. In the first part of the task, the subjects were presented with a discrete 7 × 7 regular lattice on a screen (Figure 4, upper panel on the left). The patches were linked through bonds connecting them only to neighbor patches (4 paths per node, except for the boundaries, where paths were only 2 or 3). However, we removed some of the bonds between nodes (20% of them, always preventing isolated regions in the structure from being formed) in order to introduce some level of heterogeneity in the lattice (Figure 4, left column).

The subjects were asked to visit the maximum number of patches of the resulting lattice within 49 moves if they started from the center of the structure (one step was defined as a transition between connected nodes in the graph). They were not required to complete the trajectory in any given time, so time constraints were not present in the task, and information processing could be extended as much as desired by the subject. They could move to neighbor nodes in the lattice by clicking with the mouse on the patch to which they wanted to move next (Figure 4, middle columns, showing some realizations of the resulting trajectories). Heterogeneity in the lattice then made the process non-trivial (for a homogeneous regular lattice, the optimal strategy would be simply to perform a ladder-like trajectory until all nodes were covered).

To facilitate visualization of the options available upon each decision, the current node of the individual was depicted in a different color (green, with the rest of the nodes appearing in blue), and the possible moves available at each moment were emphasized (with thicker solid lines). On the contrary, the subjects had no visual guides to distinguish between previously visited and non-visited patches, so they could only use their memory skills to avoid overlaps and increase their performance.

To assess the subjects’ performance under different levels of difficulty, the nodes in the rectangular lattice were then visually reorganized in a circular way. In one case (circular ordered), in the circle, we kept the order of the rows of the first rectangular graph (Figure 4, middle row). For the other (circular disordered), we placed the nodes according to a circular structure, but with random reorganization of nodes (Figure 4, lower row). We remark that, topologically, the three structures were completely identical, but visually different. Additionally, we rotated the rectangular structure by 90${}^{\circ }$, 180${}^{\circ }$, and 270${}^{\circ }$ (with the corresponding circular-ordered and circular-disordered reorganizations) to randomize the task (so, 12 cases in total, all with the same topological structure, were presented to each subject). The final dataset then comprised 216 trajectories with a mean duration of 77.1±2.9 s each.

As a proxy for information prospection during the task, we used eye fixations measured with a commercial eye tracker (Tobii X2-30, at 30 Hz). An eye fixation corresponded to a visual gaze on a single location on the screen (see the right column in Figure 4 for a visual trajectory example for each structure). We used this to analyze (i) the number of nodes at which the subject gazed between consecutive steps and (ii) the time for which they continued to gaze at particular patches. For this, each node was assumed to be represented by a circle of radius 0.05 (once the screen size was normalized to 1) around the center of the node, so all eye fixations lying within the circle were assumed to indicate that the subject was gazing at that particular node. This circle size prevented the assignment of fixations of the subject on different nodes at the same time.

### 5.2. Payoff Estimation from Experimental Trajectories

**Figure 8:**
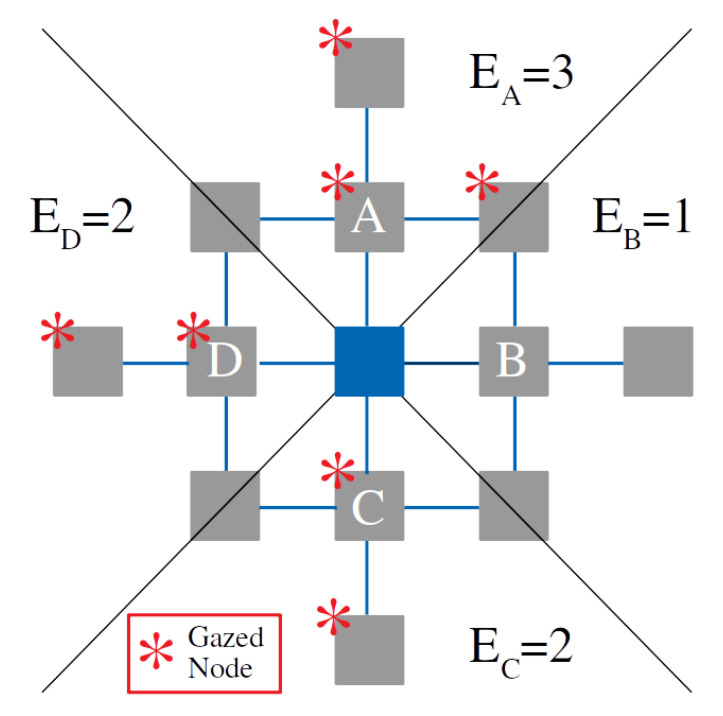
Schematic representation of how to estimate evidence from experimental gazes. The asterisks denote eye fixations, so all fixations lying in the same quadrant of one option (e.g., option A) provide evidence in favor of that option.

Using the square lattice as an example, at each time step, we divided the lattice into four equivalent regions starting from the current node (see Figure 8), so that each eye fixation that lay in a particular region would be assumed to contribute to the update of the payoff of the corresponding option (A, B, C, or D). If the individual, for example, was gazing for some time at the region corresponding to option A, then these samples were used to update the payoff $E_{A,n}$ according to the rule depicted in Figure 1. To do so, we generated a number of prospecting paths (of a given length $d_{p}$) proportional to the gazing time at random, and we computed the average number of newly visited sites that would result from those prospecting paths. The average overall prospecting paths obtained from $\left\{x_{1},x_{2},\ldots ,x_{n}\right\}$ for each option (e.g., option A) determined the estimated payoff (e.g., $E_{A,n}$).

For the circular lattices, we used exactly the same rule of implementation. As a result, the regions corresponding to each option (A, B, C, or D) were not regular, but could be disjoint and/or show different shapes depending on the specific configuration of the nodes.

### 5.3. Virtual Walkers with Prospection

An algorithm for generating virtual random walks with prospection over the lattice used in the experiment was proposed as a reference model against which to compare the experimental data. Our virtual walkers were able to estimate the convenience of moving to a neighbor node *j* by assigning successive values $\epsilon_{j,1},\epsilon_{j,2},\ldots$ to that node by prospecting hypothetical paths that would use that node as a starting point. So, at each time step, the walker prospected one particular path (chosen at random from all of the possible ones) of a fixed length $d_{p}$ (*prospection length*) starting from each of the neighbor nodes. The specific value $\epsilon_{j,n}$ assigned to the *n*-th prospected path for the neighbor node *j* corresponded to the fraction of non-visited nodes that the path would cover, with $\epsilon_{j,n}=1$ representing a prospected path for which all sites were still unvisited and $\epsilon_{j,n}=0$ representing a path for which all nodes had already been visited before. So, the corresponding payoff associated with that neighbor node *j* (after *n* paths had been prospected) reads $E_{j,n}=\frac{1}{n}\sum_{i=1}^{n}\epsilon_{j,i}$, in analogy with the working example discussed above.

Once the payoffs were defined, the procedure described in Section 2 could then be applied within the lattice to generate our virtual random walks. After each single prospection of one path in each direction, the walker computed the corresponding Shannon entropy $S_{n}=\sum_{i}^{n}-p_{j,i}\ln p_{j,i}$; if the computed value fell below a fixed threshold $S_{\mathrm{th}}$, the walker made the decision (that is, a move) by choosing the node with the highest probability (we checked that choosing the node according to the probabilities $p_{j,i}$ instead led to very similar results). On the contrary, if $S_{n}>S_{\mathrm{th}}$, then the prospection process continued. However, in practice, we introduced a rule such that the maximum number of prospections was limited to 100 to avoid (extremely unusual) situations in which $S_{n}$ would never decay below $S_{\mathrm{th}}$ because all options persistently exhibited very similar payoffs (this rule did not significantly modify any of the results reported here).

Distributed prospection lengths.

Assigning a constant prospection length $d_{p}$ to all of the prospected paths may seem rather unrealistic. Human subjects were expected to prospect paths with different lengths depending on the specific situation instead (complexity, number of choices available, etc.). The results in Figure 6b also support this, as the number of patches that were gazed at exhibited a variation that spanned almost one order of magnitude.

We then studied our virtual random-walk algorithm for the case in which a distribution of $d_{p}$ was introduced instead of a constant value. In particular, we tried a distribution $P\left(d_{p}\right)\propto \frac{1}{d_{p}^{\gamma }}$ (for $d_{p}\geq 1$ and with γ going from 0 to *∞*), with $\sum_{d_{p}=1}^{\infty }P\left(d_{p}\right)=1$ to guarantee normalization. The results, which are summarized in the Supplementary Materials, clearly show that the conclusions obtained were, thus, qualitatively the same as those presented for the fixed $d_{p}$ values in the main text.

Robustness of the distribution of decision times on the entropy threshold $S_{\mathrm{th}}$.

We reported above that the decision time for the walker exhibited a power-law distribution with an exponent of −3. An analysis to check that this exponent remained approximately constant independently of the memory and prospection parameters $d_{p}$ and $\tau_{m}$, as well as the threshold $S_{\mathrm{th}}$, was carried out by using our virtual random-walk algorithm. According to the results found (see the Supplementary Materials), the conclusions reached in the article remained quite robust. Only when very large or very small values of *S* were considered (which would represent the case in which decisions were either made almost immediately with barely any information gathering or in which an extremely large amount of information would be necessary to trigger a decision) did the ∼$n^{-3}$ scaling break down.

## Data Availability

The datasets used and analyzed during the current study are available from the corresponding author upon reasonable request.

## Supplementary Materials

The following supporting information can be downloaded at: https://www.mdpi.com/article/10.3390/e24121819/s1.
