# Supplementary material for: Informational Entropy Threshold as a Physical Mechanism for Explaining Tree-like Decision Making in Humans
Source: Entropy (Basel). 2022 Dec 13;24(12):1819. doi: 10.3390/e24121819 (PMC9778513; doi:10.3390/e24121819)
Supplement: Supplementary file 1 [file entropy-24-01819-s001.zip › entropy-2054698-supplementary (1).pdf]

Supplementary Material: Informational entropy thresholds as a physical mechanism to explain sequential decision-making

Javier Cristín*[1,2], Viçenc Méndez[3] and Daniel Campos[3]
[1] Istituto Sistemi Complessi, Consiglio Nazionale delle Ricerche, UOS Sapienza, 00185 Rome, Italy
[2] Dipartimento di Fisica, Universita' Sapienza, 00185 Rome, Italy
[3] Grup de Física Estadística, Departament de Física. Facultat de Ciències),
Universitat Autònoma de Barcelona, 08193 Bellaterra (Barcelona), Spain
(Dated: December 12, 2022)

I. GENERALIZATION OF THE WORKING EXAMPLE

In the Main Text we have examined the output of the ERM in an idealized working example. It consisted of a binary decision with options labeled as A and B. Here, we extend this idealized working example to a multiple decision situation with options A, B, C and D (whose actual payoffs are μ_A, μ_B, μ_C and μ_D, respectively). For this, the individual can sample successively data from the options, and from this data it can obtain estimates $E_{A,n}$, $E_{B,n}$, $E_{C,n}$ and $E_{D,n}$ of the payoffs, following the same procedure as described in the main text. To simplify, we assume that the process to generate the estimations $E_{A,n}$, $E_{B,n}$, $E_{C,n}$ and $E_{D,n}$ is such that at the i-th step, or sample, the piece of information obtained by the individual consists of four Gaussian variables ϵ_{A_i}, $\epsilon_{B,i}$, $\epsilon_{C,i}$, $\epsilon_{D,i}$ with corresponding means μ_A, μ_B, μ_C, μ_D respectively, and unit variance. Then, the information obtained provides an approximation to the actual values μ_A, μ_B, μ_C, μ_D and the estimated payoff can be computed through the average over the information sampled to date, so $E_{j,n} = \frac{1}{n}\sum_{i=1}^{n} \epsilon_{j,i}$, with $j = A, B, C, D$.

Once the estimated payoffs are available, we can successively compute the Shannon's entropy over the probabilites $P_{j,i}$ after i samples have been carried out, and explore the decision dynamics controlled by the termination threshold S_{th}.

We explore the statistics of decision times, this is, the number of samples n that the individual requires to reach the entropy threshold. For this, we carry out numerical experiments using the rules above and determine the distribution of decision times one typically finds when the ERM is used. The results in figure 1 confirm that the multi-optional case also reports the same exponent -3 for multiple distances between the Gaussians means d that was reported for the binary case in the Main Text.

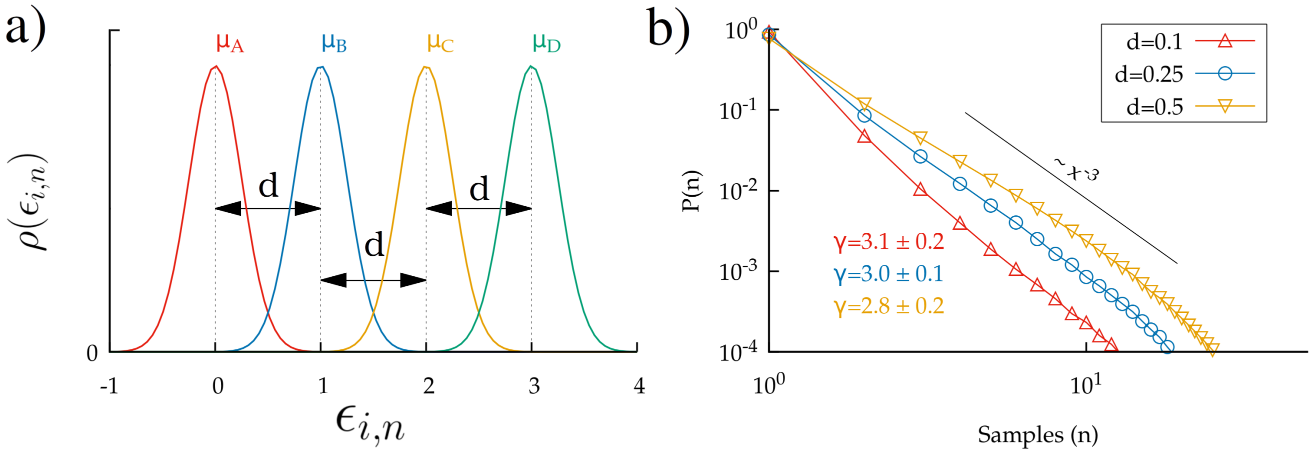

figure s1. *a) Probability distributions for the stochastic variables $\epsilon_{j,i}$, with $j = A, B, C, D$. The means $\mu_{A,B,C,D}$ correspond to the real value of each option A, B, C and D. b) Probability distribution for the number of of necessary samples (n) to reach the corresponding entropic threshold for different different distances between the means d. We have simulated 10^7 decisions to obtain those distributions.*

II. PROSPECTIVE ALGORITHM

A. Definition

We propose an algorithm in which virtual subjects are able to prospect the paths available within d_p steps in the lattice (we call this parameter the prospection length). For each path prospected, the walker assigns a payoff E_i to the neighbour node at which that path starts (for a simple visualization, see Fig. 2). The payoff is taken to be equal to the fraction of visited nodes that the prospected path crosses (so E is bounded between 0 and 1, with $E = 1$ for a path that does not cover any visited patches, and $E = 0$ if all patches covered by the path have been previously visited).

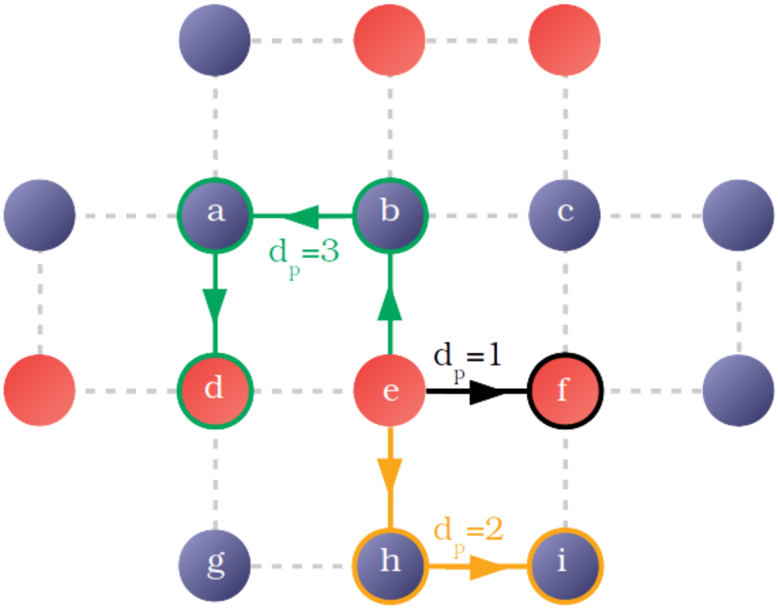

figure s2. *Scheme of three different prospection paths corresponding to three different prospection lengths $d_p = 1$ (black), $d_p = 2$(yellow) and $d_p = 3$ (green). The patches that are already visited are marked in red (so they add 0 to the payoff), while theunvisited ones appear in blue (so they add 1 to the payoff). The payoff assigned in each one of the paths depicted would be a)$E_f = 0$ to option f for $d_p = 1$, b) $E_i = (1+1)/2 = 1$ to option h for $d_p = 2$, and $E = (1+1+0)/3 = 2/3$ to option b for$d_p = 3$.*

The walker keeps in memory its previous trajectory during a characteristic number of steps. In particular, the visits are remembered by the virtual subject during a time τ obtained from the exponential distribution $P(\tau) = \frac{1}{\tau_m} e^{-\frac{t}{\tau_m}}$, with τ_m then representing the characteristic timescale of memory. After this time, the walker will forget that this particular patch has been previously visited and will contribute as a non-visited patch for computing the corresponding payoffs.

As a result, the algorithm will assign lower payoffs to the options that it remembers having visited and/or that are adjacent to regions that it remembers having visited. So, according to equation 2 in the Main Text, the probability to choose those options will decrease, so leading the virtual subject to regions that are still unvisited (or at least it does not remember having visited before). A larger prospection length d_p allows the walker to sample the state of further regions and to compute the payoff the information of distant patches, but this will be only efficient if the memory parameter τ_m is large enough.

Successive prospections of the paths available in each direction are carried out at random among all possible ones of length d_p, and so values of the payoffs and the probabilities p_i are continuously updated. Note that for a given value of d_p the number of paths that can be prospected is of the order of $\sim 4^{d_p}$ (if assuming that all bonds between neighbours are available). The algorithm makes the virtual walker to move to one of the available nodes according to the decision criterion described in the Main Text. After each single prospection of one path in each direction, the Shannon's entropy $S = \sum_i -p_i \ln p_i$ is computed; if the value falls below a fixed threshold S_{th}, the walker makes a move towards the option i satisfying $\max_i [p_{i,n}]$ computed at that time (we have checked to decide randomly according to the

probabilities does not change qualitatively the walker dynamics). On the contrary, if $S > S_{th}$ then the prospection process continues. However, we additionally introduce a rule such that the maximum number of prospections is limited to 100 to avoid (extremely unusual) situations in which S would never decay below S_{th} because all options available persistently exhibit very similar payoffs. We have carefully checked that this rule doesn't modify any of the results reported in a significant way.

B. Coverage time study

The results of the algorithm shown in the Main Text have been obtained under the same conditions that in the task presented to the human subjects; this is, for 49-step trajectories through the 7×7 lattice with the same topological structure as presented in Fig. 3 in the Main Text. However, for the sake of completeness we also analyze here the dynamics of the prospective algorithm when removing the limitation of 49 moves, and measuring instead the number of moves it takes to cover all the patches. This gives us an additional insight about the navigation efficiency of the algorithm as a function of the memory and prospection parameters, τ_m and d_p. In particular, we study the mean coverage time (T_{Cov}) (this is, the mean time required to cover all sites in the lattice). Minimization of this magnitude would then give an estimation of the navigation efficiency of the algorithm.

The main conclusion we can extract (as one can deduce from the results in Fig. 3) is that the ability to prospect future paths (so, having a large d_p) is useless unless the individual has good memory skills (this is, a large τ_m value in our context). This makes clear sense, as when the walker cannot remember the previously visited patches (low values of τ_m), the optimal strategy consists of removing prospection ($d_p = 1$); in that case the information provided by further patches represents just useless noise as the walker always sees them as non-visited patches. On the other side, for large τ_m the walker can correctly identify the previously visited patches (large values of τ_m), so then progressively higher prospection lengths d_p are found to optimize the coverage of the structure and the search of a target.

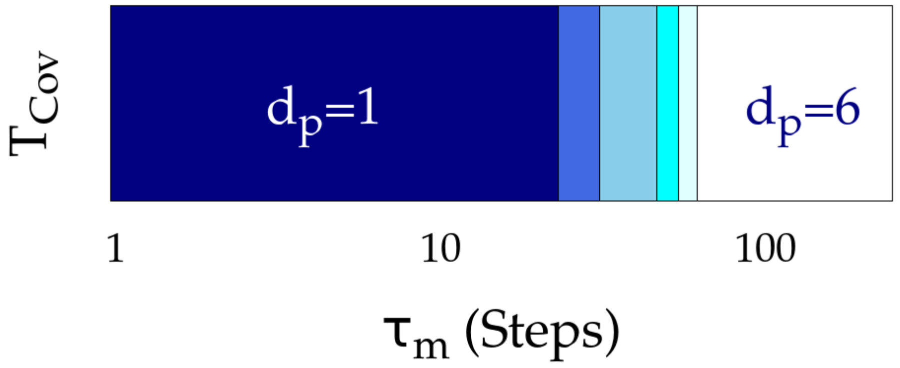

figure s3. *Prospection length d_p that minimizes the coverage process, i. e., the parameter that provides a performance thatminimizes then mean coverage time (T_{Cov}) for different values of the memory time τ_m. The data corresponds to the averagedbehavior of the walker after performing 10^4 simulations.*

C. Distributed prospection lengths

Assigning a constant prospection length d_p to all the prospected paths may seem rather unrealistic. Human individuals are expected instead to prospect paths with different lengths depending on the specific situation (complexity, number of choices available, etc). The results reported in fig 5 b) in the Main Text also support this statement (the number of gazed patches is not fixed to a constant number but exhibits a variation which spans almost one order of magnitude).

We have studied then our algorithm for the case when a distribution of d_p is introduced instead of a constant value. We have tried in particular a distribution $P(d_p) \propto \frac{1}{d_p^\gamma}$ (for $d_p \geq 1$), with $\sum_{d_p=1}^{\infty} P(d_p) = 1$ to guarantee normalization. For $\gamma \to \infty$, the paths are then fixed to $d_p = 1$, so the prospection algorithm is only to identify whether the neighbour nodes have been visited or not. On the other side, for $\gamma \to 0$ the probability is uniformly distributed among all d_p values (at practice we limit d_p to $1 \leq d_p \leq 6$ since much larger values would be absurd, given the 7×7 maze we have used). Figure 4 reports that sampling a small (but not negligible) number of long paths combined with a majority

of short paths (as happens for intermediate γ values) is sufficient to recover the results obtained for a large fixed d_p value. This can be seen by comparing (Figs. 4a and 4b) results obtained for lower values of γ to those of large values of d_p, which are extremely similar. This result is remarkable from an evolutionary perspective, since it suggests that improving navigation efficiency would not necessarily require to process much more information continually (note that the number of paths available for prospection grows in general as n^{d_p} for a sequential decision task in which n choices are given to the subject at any step, so processing costs grow exponentially with d_p). Instead, having the ability to carry out longer prospections and use this ability just promptly would be enough to increase efficiency significantly.

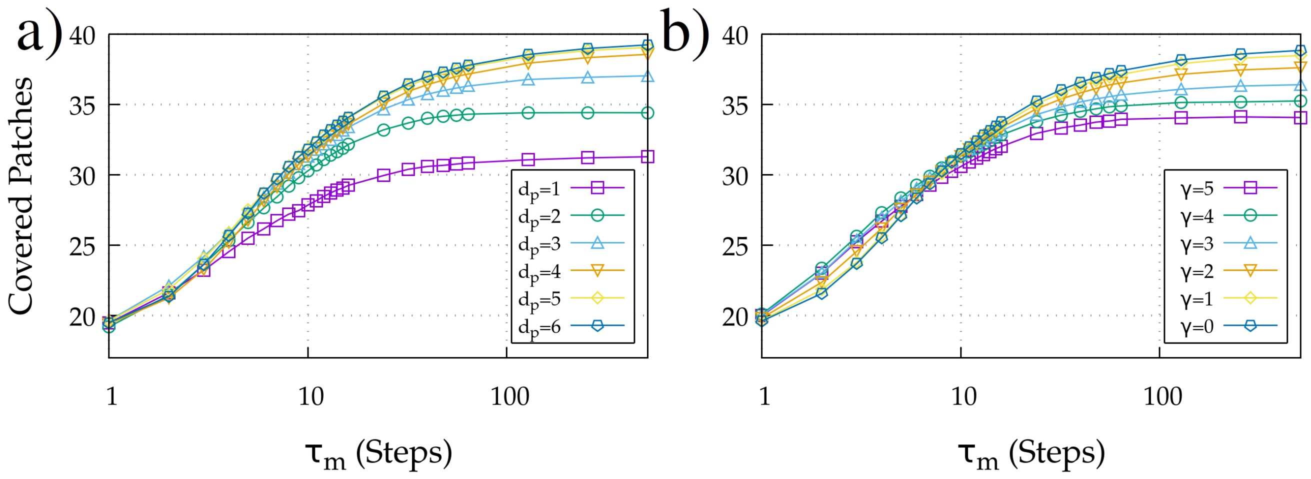

figure s4. *Averaged total number of covered patches after the 49 steps trajectory as a function of the memory time τ_m for the Virtual Walker. The graph a) corresponds to a walker prospecting with a fixed for prospection length d_p and the graph b), to awalker prospecting with a variable prospection d_p obtained from a power-law distribution with exponent γ. The data corresponds to the averaged behavior of the walker after performing 10^4 simulations.*

By exploring the whole range of γ and τ_m values, we can divide the parameter phase space into four regions (figure 5 b)), as shown in the Main Text for the case of fixed d_p. The region I produces an averaged performance that visits less patches than the individuals in any of the experimental graphs. The region II produces a performance which lies between the results obtained between Circular Ordered and Disordered. The region III overcomes the results for the Circular Ordered performance but not for the Rectangular. The region IV, finally, outperforms all the experimental results. The regions are equivalent to the obtained for fixed path lengths. Again, this shows that distributed values of d_p can be used to obtain higher navigation efficiencies without consuming much higher times of information processing.

D. Robustness of the power-law exponent for the distribution of decision times

We have reported in the Main Text that the decision time for the walker, defined as the number of samplings that are made to reach the entropy threshold S_{th}, exhibits again the same power-law distribution (with exponent -3) as the Gaussian working example. The results in Fig. 5 in the Main Text correspond to the values t_m and d_p obtained from fits to the experimental data. Here we provide an analysis to check that the -3 exponent remains as a characteristic feature of the algorithm, independent of the memory and prospection parameters, as well as the threshold S_{th} used in the algorithm.

First, in Fig. 6a we show the explicit dependence on the entropy threshold, and verify that the power-law behavior is kept as long as reasonable values of this parameter are chosen (extreme choices, with, $S_{th} \to 0$ for example, would modify the results, but we stress that this represents a rather unrealistic case for the purposes here). While the behavior of the walker is equivalent for different S_{th}, we fix it in our results in the Main Text to $S_{th} = 0.5$ so it can be conveniently applied to all choices in our maze, regardless the algorithm has two, three or four options available at that movement. On the other side, we observe at Fig. 6b that neither variations in d_p nor τ_m modify significantly the $\sim n^{-3}$ behavior as long as some significant level of memory and prospection is kept.

We stress that the classical SPRT criterion, as well as other variations we have numerically explored, are unable to reproduce the -3 exponent and would lead to much smaller exponents and/or faster (exponential-like) decays in $P(n)$. This, together with the robustness analysis reported here, provides significant robustness to the entropy threshold criterion proposed here.

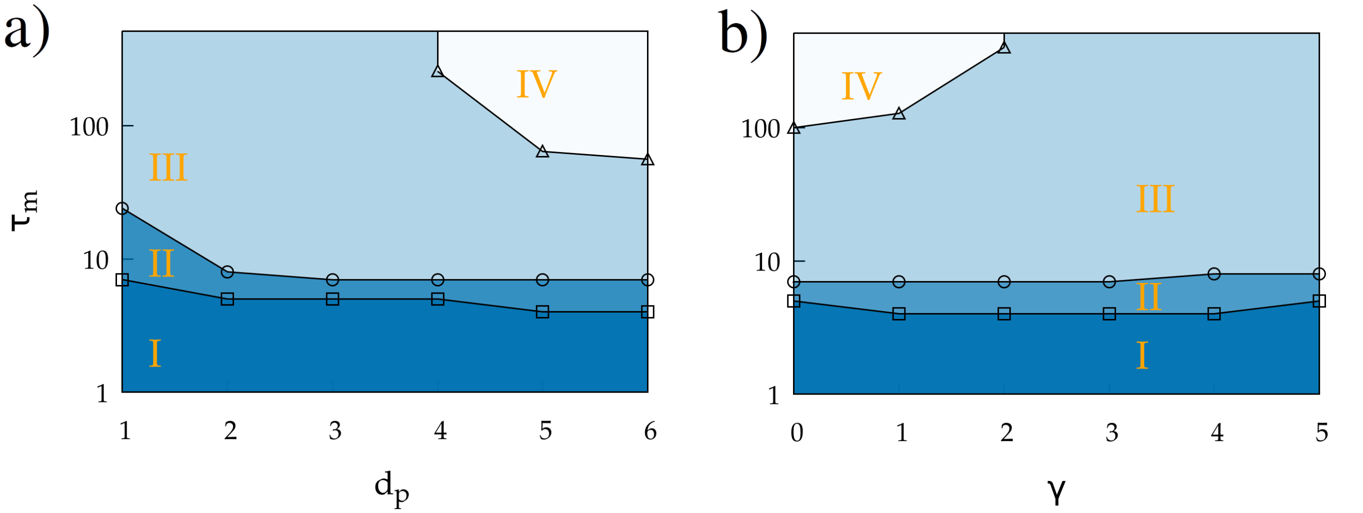

figure s5. *Diagram for the walker covered patches in comparison with experimental results. Regime I corresponds to a worse aver-aged performance than all geometries. Regime II corresponds to a better performance than in Circular Disordered. Regime IIIcorresponds to a better performance than in Circular Ordered and Disordered. Regime IV corresponds to a better performancethan in all geometries. The graph a) corresponds to a walker prospecting with a fixed for prospection length* d_p *and the graphb), to a walker prospecting with a variable prospection* d_p *obtained from a power-law distribution with exponent* γ.

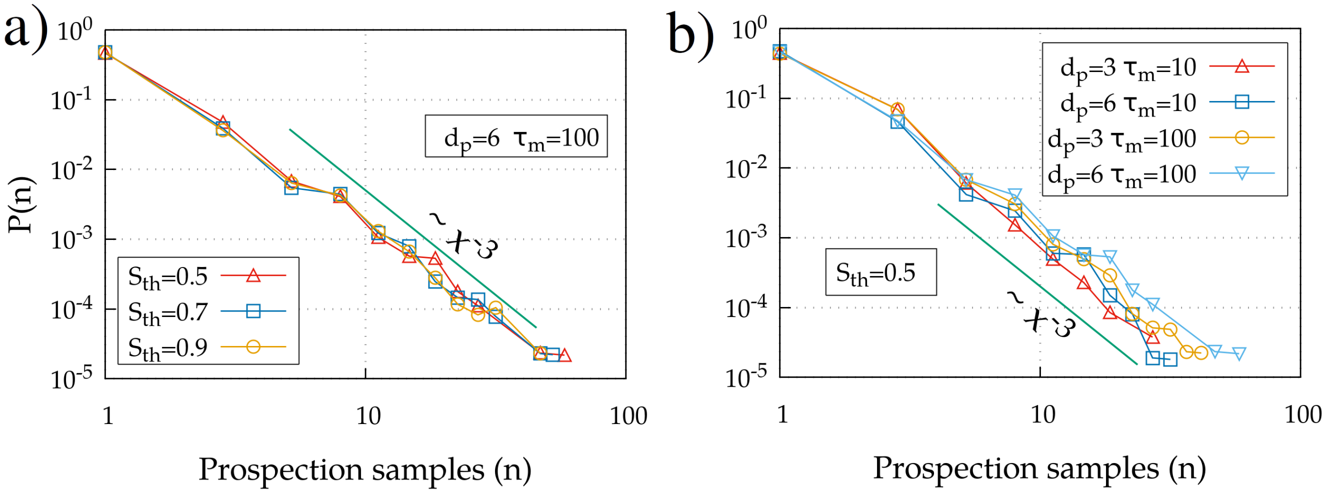

figure s6. *a) Distribution of the number of prospections n performed by the walker to force the entropy S to fall below the threshold*S_{th}. *The parameters* d_p *and* τ_m *are fixed to* $d_p = 6$ *and* $\tau_m = 100$*, respectively, while different* S_{th} *are explored. b) Distributionof the number of prospections n performed by the walker to force the entropy S to fall below the threshold* S_{th}*.The parameter*S_{th} *is fixed to* $S_{th} = 0.5$*, while different* d_p *and* τ_m *are explored. The data corresponds to the averagedbehavior of the walkerafter performing* 10^4 *simulations.*

We have also verified that the results are robust throughout the different moments of the experiment (the exponent -3 does not depend of this). This is to be expected for our experiment. For the Rectangular case, the visual complexity is low and users do not change their approach throughout the experiment. In the Circular cases, however, the fact that the memory is so short makes that, after a small number of steps, the individual resets his/her strategy.

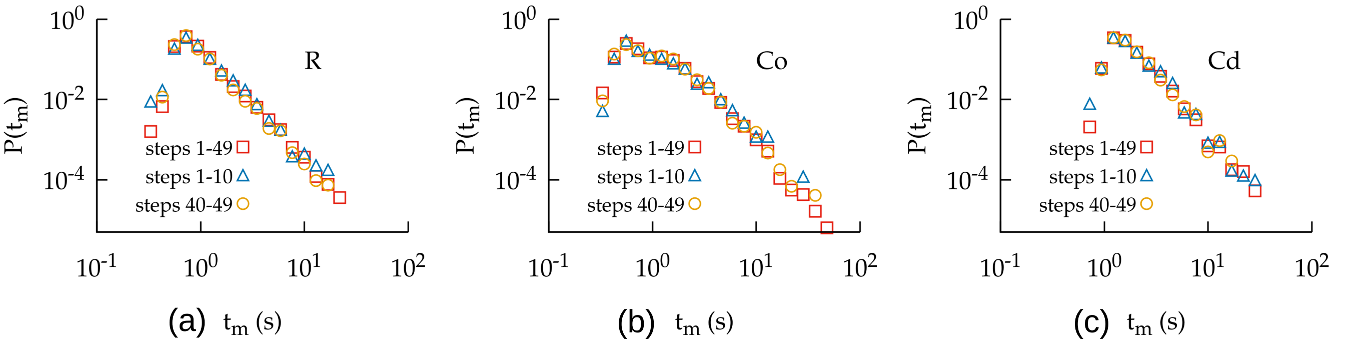

figure s7. *a) Distribution of time between consecutive moves t_m for the Rectangular case. b) Distribution of time between consecutive moves t_m for the Circular Ordered case. c) Distribution of time between consecutive moves t_m for the Circular Disordered case.*
